# Inhibition or Promotion?–The Effect of Agricultural Insurance on Agricultural Green Development

**DOI:** 10.3389/fpubh.2022.910534

**Published:** 2022-07-22

**Authors:** Dainan Hou, Xin Wang

**Affiliations:** ^1^School of Business, Minnan Normal University, Zhangzhou, China; ^2^College of Life Science, Longyan University, Longyan, China; ^3^Fujian Provincial Key Laboratory for the Prevention and Control of Animal Infectious Diseases and Biotechnology, Longyan, China; ^4^Key Laboratory of Preventive Veterinary Medicine and Biotechnology (Longyan University), Longyan, China; ^5^Chinese International College, Dhurakij Pundit University, Bangkok, Thailand

**Keywords:** agricultural insurance, agricultural green development, crowding out effect, grain functional area, China

## Abstract

Based on China's provincial panel data from 2007 to 2019, this article discusses the impact of agricultural insurance on agricultural green development, and discusses the issue of regional heterogeneity. This article first studies the impact mechanism of agricultural insurance on agricultural green development, calculates the agricultural green development index, and empirically analyzes the impact of agricultural insurance on agricultural green development. The empirical results show that agricultural insurance has an inhibitory effect on agricultural green development, and that the impact of agricultural insurance on agricultural green development in the three functional areas is heterogeneous. Finally, it puts forward countermeasures and suggestions to build a low-carbon subsidy mechanism for agricultural insurance, enrich agricultural insurance products, improve the coverage of agricultural insurance, and build an agricultural production mode of internal planting and breeding combined with recycling through policy incentives.

## Introduction

The purpose of this article was to discuss the impact of agricultural insurance on agricultural green development, and to discuss whether there is spatial heterogeneity in this impact. Since the reform and opening up, China's agricultural production has developed rapidly, relying on <10% of the cultivated land to feed approximately 20% of the world's population (1). At the same time, with the rapid development of agriculture, chemicals such as pesticides, fertilizers, and agricultural mulching film applied in agricultural production guarantee the output of agricultural and livestock products, but harm agriculture and the rural environment, and seriously affect the quality of agricultural and livestock production (2). The Chinese government attaches great importance to green development. The Fifth Plenary Session of the 18th CPC Central Committee held in 2015 incorporated the construction of ecological civilization into the overall layout of China's development. The State guided farmers to carry out green planting and breeding through policy guidance, technical support, and other measures. According to the 2016–2019 national statistical bulletin on ecological environment, the discharge of pollutants from agricultural wastewater decreased year by year from 2016 to 2019 (including chemical oxygen demand decreased from 571,000 tons in 2016 to 186,000 tons in 2019; ammonia nitrogen decreased from 13,000 tons in 2016 to 4,000 tons in 2019; total nitrogen decreased from 41,000 tons in 2016 to 13,000 tons in 2019; total phosphorus decreased from 6,000 tons in 2016 to 3,000 tons in 2019).

As an important policy tool to transfer agricultural risks and compensate farmers for disaster losses, agricultural insurance is also a green box policy allowed by WTO, which is applied by most countries in the world. Since China launched the agricultural insurance subsidy policy of the central government in 2007, agricultural insurance has achieved leapfrog development under the continuous promotion of the policy and the strong support of the government. In 2020, China's agricultural insurance premium income was RMB 81.493 billion yuan, providing RMB 4.13 trillion yuan of risk guarantee for farmers, and the compensation expenditure reached RMB 61.659 billion yuan. The national finance at all levels undertook RMB 60.3 billion yuan of guarantee fee subsidies, and the use of central government subsidy funds was increased by 145 times (3). Agricultural insurance has a certain impact on agricultural production (4) and farmers' income (4–6). It has played an increasingly important role in promoting modern agricultural development, ensuring national food security (7). It has become an important means to boost modern agricultural development, break through rural financial bottlenecks, and innovate rural governance (8).

With the rapid development of agricultural insurance, its impact on the ecological environment and agricultural green development has attracted much attention. Studies have shown that agricultural insurance can not only achieve the policy goal of dispersing agricultural production risks, but also affect farmers' production behavior. For example, Goodwin et al. (9) found that in some cases, the increase in insurance project participation will lead to statistically significant area response, although in each case, the response is very mild. Jerry (10) found that the expansion of crop insurance plans in the United States led to a significant expansion of planting area. Horowitz et al.'s (11) study showed that farmers who buy insurance tend to use relatively more chemical inputs than farmers who do not buy insurance. Smith et al. (12) had the opposite result, that is, moral hazard incentives will lead to insured farmers using fewer chemical inputs. Then, in the process of agricultural insurance, will it have an impact on agricultural green development? Is there spatial heterogeneity in the impact on different regions? On the one hand, it is helpful to grasp the reality of China's green agriculture and make an in-depth analysis of this problem; On the other hand, it helps to deeply understand the green effect of agricultural insurance, to more comprehensively evaluate the agricultural insurance policy, and to put forward feasible suggestions for further development of agricultural insurance.

Therefore, the development of agricultural insurance may have an impact on the green development of agriculture. Based on this, this study took China as the research object. First, theoretically, based on the mathematical relationship function between the economic system and economic structure proposed by Tjalling C Koopmans & John Michael Montias in 1971, this study analyzed the mechanism of the impact of agricultural insurance on agricultural green development (13) and explores the impact of the same. Based on the theory of regional economics, the heterogeneity of regional impact is discussed according to the three functions of China's grain production. Second, the evaluation system of the agricultural green development index was constructed, and the panel entropy method was used to calculate the green development index of Provincial agriculture in China from 2007 to 2019, and the calculated index was analyzed. Third, the empirical analysis of the impact of agricultural insurance on agricultural green development, using the fixed effect model to empirically analyze the overall data and the data onto three functional regions. Finally, the corresponding countermeasures and suggestions are put forward.

Some contributions to this article: Compared with previous studies, this study provides more valuable innovations. First, this study empirically tests whether China's agricultural insurance has an impact on agricultural green development. Different from previous studies, we describe the latent variable of agricultural green development by constructing the index system, trying to describe it more specifically and fully. In the selection of evaluation indicators, in addition to the traditional indicators such as chemical input used in the existing literature, we add indicators such as comprehensive utilization of resources, ecological and environmental protection, and “three rural” development. Second, the regional division is different from the traditional division. Regional heterogeneity is studied according to different grain functional areas. We do not use the geographical division commonly used in the literature but divide it according to the grain functional area. The main reason is that crop insurance accounts for a relatively high proportion of China's agricultural insurance at present. Taking 2017 as an example, the agricultural insurance premium income in that year was RMB 47.89 billion yuan, including RMB 34.557 billion yuan of crop insurance premium income, and the scale of crop insurance premium accounted for 72.16% of the total premium scale.

The structure of the rest of the parts of this article: Section Literature Review introduces the literature review, Section Theoretical Analysis and Research Hypothesis theoretical analysis and research hypothesis, Section Calculation of China's Agricultural Green Development Index measurement and analysis of China's agricultural green development index, Section Empirical Research: Data Sources and Variable reports data sources and variable selection of empirical research, Section Empirical Results Analysis reports empirical results and analysis, and Section Conclusions and Suggestions reports conclusions and puts forward suggestions.

## Literature Review

By analyzing the previous research literature, it was found that research on the impact of agricultural insurance on agricultural production mainly focuses on four aspects. First, the impact of agricultural insurance on agricultural output. Scholars have no unified conclusion on the research of this problem. Miranda (14) proposed to research that for most producers, regional yield insurance is more effective than individual farm yield insurance in resisting yield fluctuations. Akinrinola et al. (15) studied the implementation of the agricultural insurance plan in Ondo State, Nigeria, and found that access to credit is the only reason for farmers to participate in the insurance plan, while investment leads to the growth of output. Therefore, they proposed that some objectives of the agricultural insurance plan aimed at increasing agricultural production and access to credit was achieved. Taking Henan Province of China as an example, Liu et al. (16) found through empirical analysis that the development level of agricultural insurance and per capita planting area in various cities in Henan Province had a significant positive impact on agricultural output, and agricultural risks had a significant negative impact on agricultural output. Li et al. (17) have shown that both agricultural insurance and agricultural total factor productivity can significantly promote agricultural output. Dai et al. (18) have consistent research results. However, some scholars have reached the opposite conclusion. From the perspective of efficiency, Ma et al. (19) conducted an empirical analysis of China's inter-provincial panel data from 2007 to 2012 and found that agricultural insurance has a significant inhibitory effect on agricultural productivity, which is transmitted through agricultural technology, indicating that there are twists in China's agricultural insurance market, moral hazard, and adverse selection in the development of agricultural insurance. Taking Hubei Province of China as an example, Yuan et al. (20) empirically analyzed that the guarantee level and compensation level of policy agricultural insurance have a reverse effect on the agricultural output level of Hubei Province, and there are serious moral hazarded problems with the agricultural insurance market. Chambers et al. (21) extended Ramaswami's work to include multi-input and multi-output technology and concluded that the effects of insurance on output were ambiguous. Porrini et al. (22) found the importance of insurance variables and their positive impact on farm profitability by studying how the reimbursed value is used in farm management.

Second, the impact on agricultural insurance on the scale and structure of planting. When Ramasubramania (23) studied crop microinsurance in India, it was concluded that crop microinsurance had no obvious impact on its output. Xu et al. (24) found that agricultural insurance plays a positive role in cultivated area, agricultural material investment, and agricultural income, especially the change of cultivated land area is significant, but it does not play a significant role in increasing the proportion of grain income in total income. Zhang et al. (25) conducted an empirical analysis on the questionnaire of dairy farmers in the Inner Mongolia Autonomous Region and found that after controlling the endogenous nature of farmers' insurance participation behavior, the participation in dairy insurance policy significantly changed farmers' dairy farming decision-making behavior, helped to improve farmers' enthusiasm for dairy farming and encouraged farmers to expand the scale of dairy farming. Chen et al. (26) found that agricultural insurance has a scale effect and agricultural technology progress effect, but it reduces the proportion of the planting industry. Liang et al. (27) agricultural insurance has indeed promoted the expansion of the business scale of high-income farmers, but the low-security agricultural insurance has limited effect on the land inflow and scale expansion of large-scale farmers. Li (28) conducted research on planting insurance in the Inner Mongolia Autonomous Region and found that the insured farmers' cognition of planting insurance did not significantly affect their planting scale decisions, but the insured farmers' evaluation of agricultural insurance policies significantly affected farmers' planting scale decisions. The more satisfied the insured farmers are with the planting insurance policy, the greater the possibility of their stability and expansion. Roll (29) took the salmon breeding industry in Norway as an example and found that insurance enhanced production and efficiency and changed the input structure of utilization. Hill et al. (30) studied Bangladesh index insurance products and found that the purchase of insurance not only had an *ex ante* risk management effect on agricultural production practice but also had an impact on post-income; the role of risk management has led to the expansion of cultivated land. In addition, Falco (31) found that farms that grow more crops are unlikely to adopt insurance plans, and crop diversification can replace financial insurance to hedge the impact of risk exposure on welfare. In an analysis of Nebraska corn producers, Wu (32) concluded that insured farms are more likely to produce soybeans than feed crops.

Third, the impact of agricultural insurance on the investment of pesticides, chemical fertilizers, and other chemicals. Due to differences in agricultural insurance policies and premium subsidies among different countries, there are no consistent results in the academic circles on this issue. Some scholars have concluded that insurance has a certain inhibitory effect on the chemical input of pesticides and chemical fertilizers. Zhang et al. (33) found that the insured behavior inhibited farmers from applying pesticides, but the inhibitory effect was limited in terms of estimation coefficient. Based on the survey data of 858 farmers in Henan Province of China in 2018, Li et al. (17) found that compared with uninsured farmers, insured farmers tended to invest in fewer chemical fertilizers and pesticides in planting management. Zhang et al. (34) studied the farmers' insurance and factor allocation of vegetable planting professional villages in the advantageous production areas of facility vegetables in the Huang Huai Sea and the Bohai Sea and found that farmers' insurance will reduce the average fertilizer input per mu. Some scholars have concluded that the impact of insurance on the investment in pesticides, fertilizers, and other chemicals is different. Zhong et al. (35) showed that farmers who purchased agricultural insurance applied fewer pesticides and used more chemical fertilizers and agricultural film, but the impact on chemical fertilizer input was not statistically significant. Chen et al. (26) showed that the scale effect of agricultural insurance intensifies agricultural non-point source pollution, while the structural effect and technical effect reduce agricultural non-point source pollution, and there are regional differences in the impact of agricultural insurance on chemical input. Through specific analysis, it is concluded that the impact of agricultural insurance on environmental quality benefits the economically developed regions such as East China and Northeast China, while the economically underdeveloped regions such as South China and Northwest China suffer. Some scholars have concluded that insurance can promote the investment in pesticides, chemical fertilizers, and other chemicals. Horowitz (11) showed that insured farmers increased the application of chemical fertilizers, pesticides and herbicides to varying degrees. Chakir et al. (36) found through the empirical analysis of rape insurance in France that the insured farmers increased the application amount of agricultural chemicals. Luo et al. (37) conducted a comparative analysis of 87 large rice planting households and 263 ordinary farmers in the same area in Zhongshan City, Guangdong Province, China, and found that agricultural insurance affected the planting industry, increasing the application of agricultural chemicals. Pan (38) analyzed the survey data on rice farmers in various provinces in China and found that compared with non-insured farmers, the total amount of chemical fertilizer and inorganic fertilizer of insured farmers decreased by 1.7 to 3.7%. The total amount of organic fertilizer and the level of soil testing fertilization increased, and the increased proportion was 1.008 to 1.173%. Niu et al. (39) studied the pilot of policy agricultural insurance, which exacerbated the non-point sourced pollution of agricultural fertilizer in China. Li et al. (40) made an empirical analysis of the impact of agricultural insurance adoption on the application of chemical fertilizers and pesticides by fruit growers in Shaanxi, China. It was found that insured fruit farmers spent more on the use of chemical fertilizers and pesticides than uninsured fruit farmers. Capitanio et al. (41) studied the impact of insurance subsidy scheme for fertilizer used and planting proportion distribution and found that crop insurance usually has a positive impact on the optimal amount of nitrogen fertilizer for wheat and tomato. However, the research conducted by Wong et al. (42) on a drought-prone area in northern Ethiopia found that insurance-voucher programmed at best produced weak effects on stimulating agricultural activities in the immediate term. Hill et al. (30) found that the purchase of insurance not only has an *ex ante* risk management effect on agricultural production practice, but also has an impact on post-income; the income effect makes rice production more intensive in the dry season, and a large amount of irrigation and chemical fertilizer are used at the same time. Wu et al. (32) studied the impact of crop insurance on crop structure in the central Nebraska basin. The results show that providing crop insurance for corn will shift land from hay and pasture to corn. This adjustment of crop structure will increase a wide range of soil erosion and chemical use. It is very important to consider the adjustment of crop structure in the design of crop insurance plans.

Fourth, the impact on agricultural insurance in the adoption of new technologies. Many scholars have found that insurance can enable newer agricultural technologies to be adopted (43–45) Brick et al. (46) showed that most farmers are risk averse to South Africa, a developing country. Risk-averse farmers are more likely to choose traditional seeds. Despite insurance, they are unlikely to use high-yield varieties that need financing. Miao (47) studied the impact of crop survival on agricultural innovation under the background of climate change and found that the existence of American crop insurance plans hinders the innovation of crop drought tolerance, indicating that crop insurance may produce unexpected crowding out effect. Tan et al. (48) found in the analysis of contract agriculture that the age, education level, entrepreneurial training of the principal of the new agricultural operation subject and the operation scale, income, new agricultural business form, and agricultural insurance of the operating subject all had a positive impact on the demand for agricultural technology. Ma et al. (49) showed that the development of agricultural insurance could promote the progress of agricultural technology but would not promote the low-carbon transformation of agricultural production technology. Tang et al. (50) showed that “Bank-insurance Interactive” products could help disperse natural risks, alleviate the credit rationing faced by farmers, and effectively promote farmers' choice of new technologies. Michael et al. (51) showed that agricultural index insurance could promote the adoption of improved technologies, but the impact was limited to environments with high risks and significant covariation. Liu (52) analyzed the survey data from 219 vegetable farmers in Shandong Province by using the structural equation method. It is concluded that agricultural insurance has a strong impact on farmers' willingness to adopt technology from the aspects of technology adoption motivation and ability, and has a more obvious impact on large-scale farmers. Schoengold et al. (53) empirically analyzed the county-level farming practice data provided by conservation tillage information centers in Iowa, Nebraska, and South Dakota. The empirical results showed that recent disasters and compensation payments were related to the increase of no tillage use and the decrease of other conservation tillage use; Producers in counties with recent drought and flood disasters are more likely to use other conservation tillage; Changes in agricultural policies, such as disaster payments and crop insurance, may have an unintended impact on the use of agricultural protection measure. The research results from Tang et al. (54) showed that weather index insurance had a significant impact on farmers' technology adoption.

The existing literature has made some achievements in the impact of agricultural insurance on agricultural production, which provides a literature reference from this study. However, there is still less literature on the impact of agricultural insurance on agricultural green production and agricultural green development. First, the existing literature mainly studies one aspect of agricultural production, such as output, planting and breeding scale, planting and breeding structure, chemical input, and new technology adoption, but there is a lack of research on the overall impact on agricultural production. In this article, the agricultural green development level is included in an evaluation index to study, and its coefficient is calculated. Compared with previous studies, this study reflects the level of agricultural green development comprehensively and systematically. Second, in terms of selection of indicators to measure the development level of agricultural insurance, previous literature adopted indicators such as agricultural insurance premium income, agricultural insurance indemnity amount, or agricultural insurance density to measure the development level of agricultural insurance. These indicators are often used in agricultural production at the provincial level in China, but it is slightly insufficient to use these indicators to measure the development level of agricultural insurance.

Therefore, this article uses the per capita agricultural insurance premium income of agricultural employees in various regions as an index to measure the development level of agricultural insurance.

## Theoretical Analysis and Research Hypothesis

### Mechanism Analysis of the Impact of Agricultural Insurance on Agricultural Green Development

Tjalling C Koopmans and John Michael Montias, an American institutional economist, put forward a mathematical relationship function of the economic system and economic structure in his book in 1971, indicating that economic results depend on the environment, system, and policy (13). The mathematical expression is as follows:


(1) $$\begin{array}{l} O=f\left(E,S,P_{S}\right) \end{array}$$

O represents the economic result, E represents economic development environment, S and Ps represent the economic system and economic policy, respectively.

Agricultural insurance has the functions of transferring and dispersing agricultural risks, supplementing disaster losses and balancing farmers' income. It is an important means of agricultural risk management and is adopted by most countries in the world. Due to the quasi-public goods characteristics of agricultural insurance (55), the government plays an important role in the development of agricultural insurance. From the practice of agricultural insurance in various countries, governments support the development of agricultural insurance on tax incentives, premium subsidies, government disclosure, and other measures. However, agricultural insurance subsidies and public aid may cause the crowding-out effect of agricultural insurance (56).

**Figure 1:**
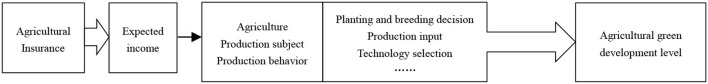
The impact mechanism of agricultural insurance and agricultural green development level.

Agricultural insurance affects the expected income of agricultural production and then affects the planting and breeding decision making of agricultural production subjects (planting and breeding scale, planting and breeding varieties, and so on), production investment (chemical investment, disaster relief investment, and others), technology selection (farming technology, adoption of new technology, and so on), which further affects the green development of agriculture (as shown in Figure 1).

Therefore, the impact of agricultural insurance on agricultural green development must pass through a certain carrier, that is, through the impact of the production behavior of agricultural production subjects, and then affect the level of agricultural green development.

Through the above analysis, this article uses the formula (1) for reference to analyze the impact of agricultural insurance on agricultural green development. By expressing the agricultural insurance policy and agricultural green development level as the economic system, economic policy, and economic results in the original formula, and retaining other variables, a new function expression can be obtained:


(2) $$\begin{array}{l} P=f\left(E,R,\mathrm{Pr}\right) \end{array}$$

In the formula, *P* represents the green development level of agriculture, *E* represents the factors affecting the green development level of agriculture, such as the environment for economic development, where *R* represents the production behavior of farmers, and Pr represents the agricultural insurance policy.

Based on the above analysis, this article puts forward hypothesis 1:

- H1a Agricultural insurance can promote the green development of agriculture.
- H1b Agricultural insurance can inhibit the green development of agriculture.

### Heterogeneity Analysis of the Impact of Agricultural Insurance on Agricultural Green Development

China has a vast territory, large longitude and latitude span, different terrain levels, various terrain types, and mountain trends. Due to different combinations of temperature and precipitation, a variety of climates have been formed. In addition, agricultural producers in different regions have different planting habits, planting concepts, and planting structures, so the development level of agricultural insurance and green agricultural development in different regions are quite different.

This article discusses the impact of crop insurance on agricultural green development from three functional areas of grain in China: main production area, main sales area, and balance area. Major grain-producing areas cover 13 provinces, including Heilongjiang, Henan, Shandong, Sichuan, Jiangsu, Hebei, Jilin, Anhui, Hunan, Hubei, Inner Mongolia Autonomous Region, Jiangxi, and Liaoning. The main grain-producing areas are mainly large grain-producing provinces, which account for more than 75% of the national grain output and shoulder the important responsibility of food security. Besides, the provinces in the main grain-producing areas attach great importance to food production, which may make the green agricultural development level in this region different from the other two functional areas. At the same time, to prevent agricultural risks, the state and major producing provinces attach great importance to avoiding agricultural risks through agricultural insurance.

In 2021, the Ministry of agriculture and rural areas, the Ministry of Finance, and the China Banking and Insurance Regulatory Commission issued the notice on expanding the implementation scope of full cost insurance and planting income insurance for the three major grain crops, gradually providing all farmers in major grain-producing counties in 13 major grain-producing provinces with insurance coverage covering the full cost of agricultural production or planting income. However, agricultural insurance is the “stabilizer” of agricultural production. It has the characteristics of both quasi-public goods and some commercial insurance. It needs some support from governments at all levels. However, large grain-producing counties are also small financial counties, and the support for agricultural insurance is limited. Therefore, in the main grain-producing areas, agricultural insurance may be beneficial or harmful to agricultural green development.

The main grain sales areas include seven provinces and municipalities directly under the Central Government: Beijing, Tianjin, Shanghai, Zhejiang, Fujian, Guangdong, and Hainan. The main grain sales areas are mainly municipalities directly under the central government and coastal provinces with a relatively developed economy, which have a good financial environment, but there are many people and less land, the self-sufficiency rate of grain is low, and there is a large demand for vegetables and other agricultural and sideline products. Therefore, there are some differences between this area and other functional areas in terms of crop planting structure and farmers' planting experience, agricultural insurance development, and government policy guidance and support. In addition, the large population may have some impact on the agricultural production environment. It can be seen that agricultural insurance in the main grain sales areas may have some impact on the level of agricultural green development, but the direction is uncertain.

The areas with balanced grain production and sales cover 11 provinces, including Shanxi, Guangxi, Chongqing, Guizhou, Yunnan, Tibet, Shaanxi, Gansu, Qinghai, Ningxia, and Xinjiang autonomous region. Most of the provinces in the balanced grain production and sales areas are located in western China, and their economic development is slightly weaker than that of other functional areas. Agricultural production conditions, such as natural conditions and soil conditions, are relatively poor. However, governments at all levels should attach great importance to agricultural production and take multiple measures to promote agricultural production and income. For example, Guizhou Province and Yunnan Province ensure the high yield of corn through measures such as high-quality varieties, machine tillage and sowing, black film mulching, interplanting potato and beans, deep tillage and soil loosening, and green fertilizer planting. One has to take advantage of the climate to vigorously promote the industrialized planting of potatoes. Therefore, agricultural insurance on grain production and sales balance area has some impact on the level of agricultural green development, and the degree and direction of impact are uncertain.

Based on this, this article puts forward hypothesis 2:

- H2a The impact of agricultural insurance on agricultural green development has regional heterogeneity.
- H2b There is no regional heterogeneity in the impact of agricultural insurance on agricultural green development.

## Calculation of China'S Agricultural Green Development Index

### Building an Evaluation Index System

It is a complex project to build an evaluation index system of China's agricultural green development level. The index system should be systematic, guiding, and scientific, and the selected index should be comprehensive and operable. In recent years, the Chinese government has attached great importance to agricultural green development and issued a series of guiding documents, which provides a theoretical basis for a deep understanding of the connotation of agricultural green. Based on policy research, theoretical research, and previous research (57), this article constructs an evaluation index system consisting of four dimensions and 12 indicators (see Table 1).

**Table 1:** The evaluation index system of agricultural green development level.

**Primary index**	**Secondary index**	**Unit**	**Computing method**	**Index attribute(+ or -)**
Comprehensive utilization of resources A	Unit planting area yield of grain A1	Ton / ha	Grain yield / grain sowing area	+
	Unit planting area total agricultural output value A2	RMB 10,000 yuan / ha	Total agricultural output value / sown area of crops	+
	Effective irrigation efficiency A3	%	Water saving irrigation area / grain sowing area	+
Ecological environment protection B	Percentage of forest cover B1	%	Directly from the yearbook	+
	Areas of soil erosion under control B2	1,000 ha	Directly from the yearbook	+
Agricultural chemicals input C	Pesticide application intensity C1	Ton / ha	Application amount / grain sowing area	–
	Pesticide application intensity C2	Ton / ha	Fertilizer application amount / grain sowing area	–
	Pesticide application intensity C3	Ton / ha	Application amount of agricultural film / grain sowing area	–
“Three rural” development D	Per capita agriculture, forestry, animal husbandry and fishery GDP D1	RMB yuan / person	Total output value of agriculture, forestry, animal husbandry and fishery / agricultural population	+
	Quantity of green food per unit area D2	PCs. / ha	Number of green food certification / grain sowing area in that year	+
	Engel coefficient of rural residents D3	%	Yearbook direct access	–
	Unit planting area total power of agricultural machinery D4	KW / ha	Total power of agricultural machinery / grain sowing area	+

### Agricultural Green Development Index Measurement Method

There are many methods of weight calculation. For example, Pourjavad et al. (58) use a fuzzy multi criteria decision-making (MCDM) approach to obtain weights. Tseng et al. (59) calculate the weight through the Fuzzy Delphi method. In this article, the entropy method is used to calculate the agricultural green development index. This method belongs to the objective weighting method, which can effectively avoid the weight deviation caused by subjective error.

The calculation steps of panel data entropy method are as follows:

Step 1: index standardization. There are great differences in units and orders of magnitude among different indicators. To ensure the reliability of evaluation results, the original data need to be standardized before calculation and comparison. The calculation formula is as follows:

Positive index: $X_{ijt}^{*}=\frac{x_{ijt}-\min\left(x_{i}\right)}{\max\left(x_{i}\right)-\min\left(x_{i}\right)}$ (1)

Negative index: $X_{ijt}^{*}=\frac{\max\left(x_{i}\right)-x_{ijt}}{\max\left(x_{i}\right)-\min\left(x_{i}\right)}$ (2)

Where, x_ijt_ and X${}_{\text{ijt}}^{{}^{*}}$ respectively, represent the original value and standardized value of index *j* in the *t* year of the *i* province, max (x_i_) and min(x_i_) are the maximum and minimum values of index j in the sample period of 30 provinces and regions, respectively.

Step 2: calculate the weight W_ij_ of each index by entropy weight method. The principle of entropy weighted method is to obtain the information entropy of each index according to the inherent information on each scheme for the evaluation. The greater the information entropy, the smaller the utility value of information and the smaller the index weight. The calculation process is as follows:

Calculate the proportion of the *j* index of the *i* Province in the *t* year:


(3) $$\begin{array}{l} Y_{ijt}=\frac{X_{ijt}^{*}}{\overset{n}{\underset{i=1}{\sum }}X_{ijt}^{*}},i\in \left[1,n\right],j\in \left[1,m\right] \end{array}$$

Calculate the information entropy of the *j* index in the *t* year:


(4) $$\begin{array}{l} e_{jt}=-M\times \overset{n}{\underset{i=1}{\sum }},\left(Y_{ijt}\times \ln\;Y_{ijt}\right),\;M=\frac{1}{\ln\;\left(n\right)} \end{array}$$

Calculate information entropy redundancy:


(5) $$\begin{array}{l} d_{jt}=1-e_{jt} \end{array}$$

Calculation of index weight:


(6) $$\begin{array}{l} W_{jt}=\frac{d_{jt}}{\overset{m}{\underset{j=1}{\sum }}d_{jt}} \end{array}$$

Step 3: development index calculation. First, calculate the standardized scores of 12 indicators in a certain year(X${}_{\text{ijt}}^{{}^{*}}$). Second, the weight of each index is multiplied by the standardized score of the corresponding index(X${}_{\text{ijt}}^{{}^{*}}\times$W_j_). Finally, the weighted scores of each index are summed to obtain the score of the agricultural green development index of each province, that is, $F_{i}=\overset{12}{\underset{j=1}{\sum }}\left(X_{ij}^{*}\times W_{j}\right)$. Repeat this calculation step for different years.

### Calculation Results and Trend Analysis of Agricultural Green Development Index

After the above calculation steps, calculate the weight of each index from 2007 to 2019, as shown in Table 2. According to the formula, the agricultural green development index of China's provinces from 2007 to 2019 is calculated and reported in Table 3. According to the three different functional areas, the average scores of the agricultural green development index of each province in the three regions in this range are listed and arranged in the order from high to low, which is reported in Table 4. The areas with a high agricultural green development index are mainly concentrated in the main grain sales areas and main grain production areas. There are six provinces with agricultural green development index ≥0.3 in the main grain sales areas, namely, Beijing, Zhejiang, Fujian, Tianjin, Shanghai, and Hainan, accounting for 85.71% of the region. There are six provinces with agricultural green development index ≥0.3 in the main grain-producing areas, namely, Liaoning, Inner Mongolia, Shandong, Jiangsu, Hebei, and Heilongjiang, accounting for 46.15% of the region. The agricultural green development index of the grain production and sales balance region is ≥0.3, only Tibet Autonomous Region, accounting for 9.09% of the region. It can be seen from the above analysis data that grain main sales areas have some advantages in agricultural green development, while grain production and marketing balance areas are in a relatively weak position. Through the analysis of specific indicators, it is found that the main grain sales areas have some advantages in indicators such as total power of agricultural machinery per unit area, forest coverage, and per capita water and soil control area. The reason may be that the three functional areas have great differences in geographical location, functional area division, agricultural industrial structure, and local policies.

**Table 2:** The weight of each index (2007–2019).

**Year**	**W_A1**	**W_A2**	**W_A3**	**W_B1**	**W_B2**	**W_C1**	**W_C2**	**W_C3**	**W_D1**	**W_D2**	**W_D3**	**W_D4**
2007	0.0,554	0.0,996	0.0,813	0.0,916	0.1,505	0.0266	0.0400	0.0,285	0.0,615	0.1,983	0.0,419	0.1,248
2008	0.0,617	0.0,996	0.0,918	0.0,896	0.1,462	0.0,188	0.0,443	0.02,49	0.0,596	0.1,781	0.0,709	0.1,144
2009	0.0,496	0.1,046	0.1,100	0.0,741	0.1,531	0.0,140	0.0,536	0.0,284	0.06,24	0.2,025	0.0,412	0.1064
2010	0.0,727	0.0,992	0.1,090	0.0,740	0.1,508	0.0146	0.0452	0.0,288	0.0,683	0.1,663	0.0,705	0.1006
2011	0.0,455	0.1,206	0.1,092	0.0,790	0.1,625	0.0,155	0.0,443	0.0,383	0.0,651	0.1,690	0.0,487	0.1,022
2012	0.0,602	0.1,367	0.1,017	0.0,751	0.1,535	0.0,178	0.0347	0.0,400	0.0,583	0.1,856	0.0,334	0.1,029
2013	0.0,525	0.1,485	0.0,841	0.0,757	0.1,416	0.0,161	0.0374	0.0,394	0.0,577	0.2,090	0.0,260	0.1,120
2014	0.0,591	0.1,623	0.0,838	0.0,739	0.1,373	0.0,173	0.0320	0.0,392	0.0,563	0.2,011	0.0,182	0.1,196
2015	0.0,558	0.1,502	0.0,912	0.0,749	0.1,453	0.0,171	0.0304	0.0,342	0.0,607	0.1,931	0.0,188	0.1,283
2016	0.0,462	0.1,385	0.0,793	0.0,720	0.1,420	0.0,191	0.0279	0.0,258	0.0,652	0.2,056	0.0,175	0.1,609
2017	0.0,466	0.1,379	0.0,830	0.0,728	0.1,394	0.0,177	0.0376	0.0,207	0.0,679	0.1,968	0.0,198	0.1,598
2018	0.0,434	0.1,435	0.0„867	0.0,708	0.1,342	0.0,294	0.0379	0.0,176	0.0,683	0.2,338	0.0,256	0.1,088
2019	0.0,453	0.1,286	0.0,866	0.0,651	0.1,213	0.0,095	0.0347	0.0,147	0.0,608	0.2,891	0.0,227	0.1,215

*Data source, calculated by entropy method*.

**Table 3:** The score of agricultural green development index of 31 provinces in China from 2007 to 2019.

**Year**	**2007**	**2008**	**2009**	**2010**	**2011**	**2012**	**2013**	**2014**	**2015**	**2016**	**2017**	**2018**	**2019**
Beijing	0.6,286	0.6,281	0.6280	0.6,023	0.5,782	0.5,842	0.5,998	0.5,965	0.5,937	0.5,930	0.6,100	0.6,523	0.6,276
Tianjin	0.4,190	0.4,016	0.3,818	0.4,052	0.4,211	0.3,487	0.3,226	0.3,098	0.3,142	0.2,869	0.3,003	0.2,720	0.2,367
Hebei	0.3,562	0.3,577	0.3,375	0.3,754	0.3,802	0.3,493	0.3,201	0.2,880	0.2,835	0.2,515	0.2,658	0.2,640	0.2,311
Shanxi	0.2,610	0.2,528	0.2,395	0.2,444	0.2,466	0.2,281	0.2,289	0.2,168	0.2,139	0.1,829	0.1,760	0.1,873	0.1,591
Inner Mongolia	0.4,023	0.4,076	0.3,984	0.4,189	0.4,257	0.3,913	0.3,745	0.3,505	0.3,527	0.3,260	0.3,268	0.3,425	0.2,927
Liaoning	0.4,488	0.4,695	0.4,344	0.4,498	0.4,537	0.4,153	0.3,727	0.3,305	0.3,374	0.3,081	0.3,162	0.3,058	0.2,715
Jilin	0.3,469	0.3,678	0.3,311	0.3,527	0.3,430	0.3,206	0.2,717	0.2,549	0.2,507	0.2,359	0.2,410	0.2,314	0.2,062
Heilongjiang	0.2,943	0.3,302	0.3,094	0.3,323	0.3,363	0.3,115	0.3,030	0.2,890	0.2,894	0.2,761	0.2,884	0.2,944	0.2,482
Shanghai	0.3,008	0.3,190	0.3,020	0.3,501	0.3,561	0.3,213	0.2,969	0.2,970	0.2,864	0.2,911	0.2,979	0.3,654	0.4,707
Jiangsu	0.3,130	0.3,299	0.3,317	0.3,730	0.3,751	0.3,382	0.3,126	0.3,050	0.3,046	0.2,860	0.2,911	0.2,875	0.2,552
Zhejiang	0.4,894	0.5,199	0.5,121	0.5,666	0.5,923	0.5,323	0.5,064	0.4,773	0.4,597	0.4,389	0.4,347	0.4,064	0.3,579
Anhui	0.2,405	0.2,462	0.2,437	0.2,584	0.2,640	0.2,456	0.2,364	0.2,290	0.2,274	0.2,344	0.2,421	0.2,364	0.2,083
Fujian	0.3,886	0.4,228	0.4,173	0.4,379	0.4,688	0.4,572	0.4,733	0.4,633	0.4,681	0.4,527	0.4,529	0.4,320	0.3,952
Jiangxi	0.3,225	0.3,279	0.3,237	0.3,241	0.3,447	0.3,126	0.2,791	0.2,694	0.2,664	0.2,484	0.2,522	0.2,512	0.2,223
Shandong	0.3,371	0.3,649	0.3,560	0.3,919	0.3,866	0.3,517	0.3,272	0.3,123	0.3,077	0.2,790	0.2,921	0.2,788	0.2,469
Henan	0.2,611	0.2,725	0.2,502	0.2,769	0.2,531	0.2,429	0.2,221	0.2,129	0.2,100	0.1,936	0.2,021	0.1,984	0.1,759
Hubei	0.2,794	0.2,931	0.2,921	0.3,173	0.3,462	0.3,135	0.3,062	0.2,932	0.2,919	0.2,738	0.2,791	0.2,725	0.2,413
Hunan	0.3,079	0.3,106	0.3,018	0.3,041	0.3,175	0.2,944	0.2,818	0.2,698	0.2,663	0.2,599	0.2,721	0.2,657	0.2,484
Guangdong	0.3,051	0.3,251	0.3,169	0.3,238	0.3,448	0.3,277	0.3,145	0.3,070	0.2,997	0.2,760	0.2,663	0.2,568	0.2,229
Guangxi	0.2,489	0.2,288	0.2,414	0.2,407	0.2,701	0.2,563	0.2,556	0.2,473	0.2,434	0.2,322	0.2,373	0.2,362	0.2,066
Hainan	0.2,734	0.2,829	0.2,846	0.2,902	0.3,142	0.3,122	0.3,152	0.3,070	0.3,143	0.3,258	0.3,411	0.3,195	0.3,093
Chongqing	0.2,119	0.2,140	0.2,295	0.2,445	0.2,555	0.2,367	0.2,305	0.2,280	0.2,289	0.2,209	0.2,234	0.2,434	0.2,195
Sichuan	0.2,497	0.2,434	0.2,603	0.2,522	0.2,718	0.2,547	0.2,553	0.2,402	0.2,420	0.2,278	0.2,308	0.2,324	0.1,980
Guizhou	0.1,681	0.1,540	0.1,810	0.1,712	0.1,776	0.1,789	0.1,871	0.1,888	0.2,058	0.1,956	0.2,071	0.2,286	0.1,912
Yunnan	0.2,368	0.2,164	0.2,234	0.2,126	0.2,436	0.2,267	0.2,330	0.2,262	0.2,257	0.2,217	0.2,310	0.2,349	0.2,059
Tibet	0.3,759	0.3,886	0.3,874	0.3,836	0.3,743	0.3,654	0.3,797	0.3,862	0.3,811	0.3,790	0.3,996	0.3,531	0.3,253
Shaanxi	0.2,905	0.2,988	0.2,815	0.3,041	0.3,263	0.2,979	0.2,690	0.2,568	0.2,599	0.2,409	0.2,439	0.2,488	0.2,105
Gansu	0.2,324	0.2,296	0.2,457	0.2,315	0.2,430	0.2,170	0.2,078	0.1,878	0.1,978	0.1,816	0.2,057	0.2,168	0.1,917
Qinghai	0.3,180	0.3,467	0.3,564	0.3,578	0.3,959	0.3,215	0.2,705	0.2,479	0.2,426	0.2,405	0.2,664	0.2,869	0.2,377
Ningxia	0.2,957	0.2,958	0.2,916	0.3,116	0.3,097	0.2,872	0.2,748	0.2,750	0.2,805	0.2,690	0.2,721	0.2,789	0.2,309
Xinjiang	0.2,958	0.2,893	0.2,809	0.3,358	0.2,901	0.2,833	0.2,856	0.2,640	0.2,704	0.2,368	0.2,414	0.2,493	0.2,062

**Table 4:** The average score of the agricultural green development index in all regions from 2007 to 2019.

**Main grain** **producing areas**	**Agricultural green** **development index**	**Main grain** **sales area**	**Agricultural green development index**	**Grain production and sales balance area**	**Agricultural green development index**
Liaoning	0.3,780	Beijing	0.6,094	Tibet	0.3,753
Inner Mongolia	0.3,700	Zhejiang	0.4,841	Qinghai	0.2,991
Shandong	0.3,256	Fujian	0.4,408	Ningxia	0.2,825
Jiangsu	0.3,156	Tianjin	0.3,400	Xinjiang	0.2,715
Hebei	0.3,123	Shanghai	0.3,273	Shaanxi	0.2,714
Heilongjiang	0.3,002	Hainan	0.3,069	Guangxi	0.2,419
Hubei	0.2,923	Guangdong	0.2,990	Chongqing	0.2,297
Jilin	0.2,888			Yunnan	0.2,260
Jiangxi	0.2,880			Shanxi	0.2,183
Hunan	0.2,846			Gansu	0.2,145
Sichuan	0.2,430			Guizhou	0.1,873
Anhui	0.2,394				
Henan	0.2,286				
Mean value	0.2,974		0.4,011		0.2,561

**Figure 2:**
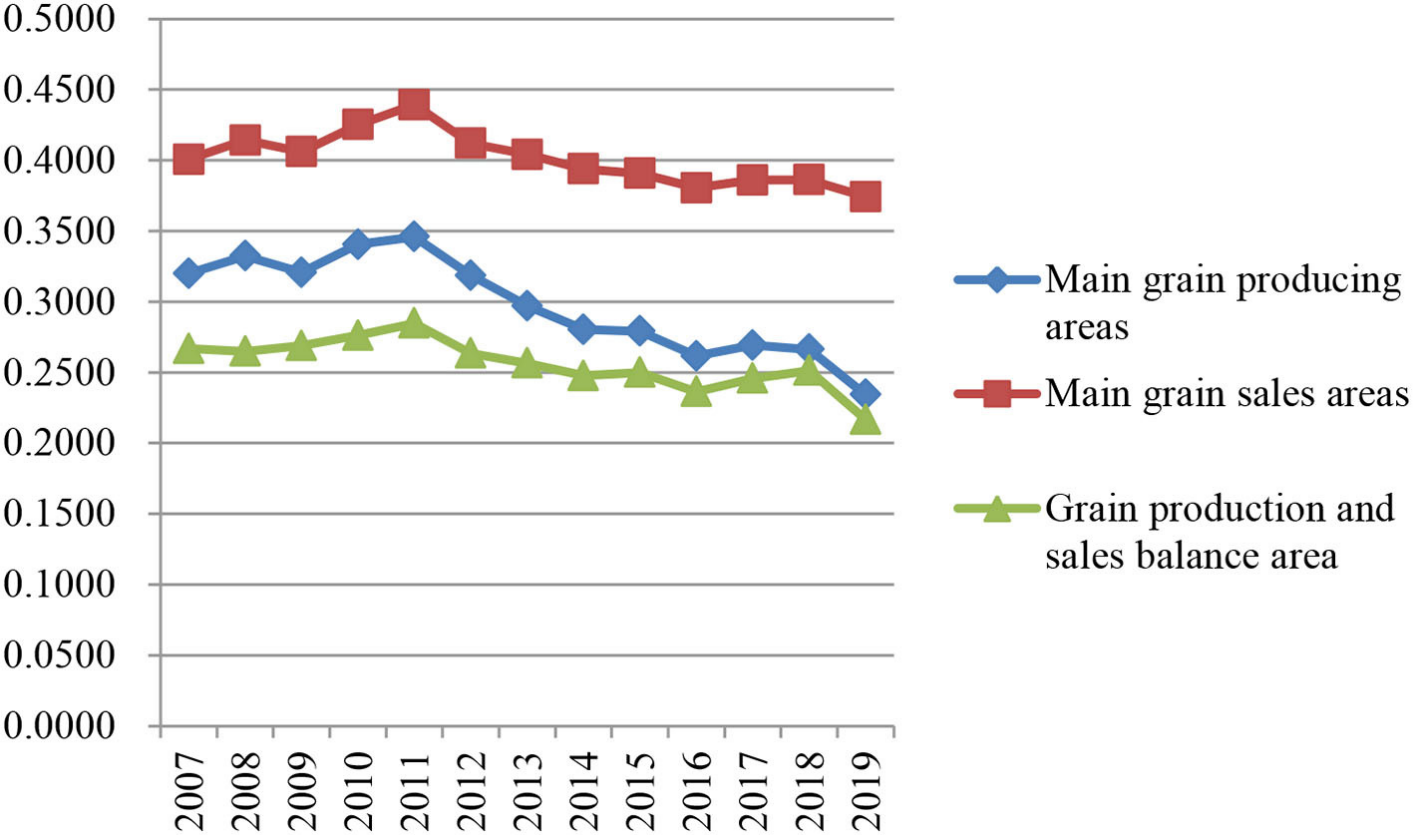
The development trend of the agricultural green development index in three functional areas of grain production in China (2007–2019).

To better to analyze the changing trend of the agricultural green development index in each district, the arithmetic average is obtained according to the agricultural green development index of provinces in each district during 2007–2019. As shown in Figure 2, in this time interval, the agricultural green development index is the highest in the main grain sales area, followed by the main grain production area and the lowest in the grain production and sales balance area. The overall trend of the agricultural green development index in the three regions is the same, with a fluctuation range of 0.2168–0.4393.

## Empirical Research: Data Sources and Variable Selection

### Data Sources

Since 2007, the Chinese government has implemented the agricultural insurance premium subsidy policy, so the research interval of this study was selected from 2007 to 2019. The data come from the China Statistical Yearbook (2008–2020), China Insurance Yearbook (2008–2020), China Rural Statistical Yearbook (2008–2020), and the statistical yearbook of 31 provincial administrative units (2008–2020). Some data are processed and calculated on the basis of original data.

### Variable Selection

The explanatory variable is the agricultural green development index. The core explanatory variable is the development level of agricultural insurance. Most previous pieces of literature evaluate and measure it through indicators such as agricultural insurance premium income (60) and agricultural insurance density (16, 61). China's agricultural production at the provincial level has its characteristics. It is slightly insufficient to measure the development level of agricultural insurance only by the total amount absolute index of agricultural insurance premium income. Although the agricultural insurance density (agricultural insurance premium income / regional agricultural population) is a relative indicator, the regional agricultural population is affected by regional urbanization and industrial structure, so it is inappropriate to take the regional agricultural population as the denominator of the calculation formula. Therefore, based on the measurement method of Zhou et al. (62), this study used the per capita agricultural insurance premium income of agricultural employees in various regions as an index to measure the development level of agricultural insurance. The indicators of control variables mainly include three indicators: first, the variables reflecting the macroeconomic situation of each province, such as the level of economic development, the gap between urban and rural areas, and the level of financial support for agriculture. The second is the variables that reflect the basic situation of “agriculture, rural areas, and farmers” in all provinces, such as industrial structure, grain output, and water-saving irrigation. Based on the above analysis, the selected variables and descriptions are shown in Table 5.

**Table 5:** Relevant variables and description.

**Variable**	**Variable abbreviation**	**Variable name**	**Acquisition method**	**Unit**
Explained variable	agdi	Agricultural green development index	Calculation acquisition	—-
Core explanatory variable	ai	Development level of agricultural insurance	Agricultural insurance premium income / number of agricultural employees in the region	RMB yuan / person
Control variable	ri	Per capita net income of rural residents	Yearbook direct access	RMB yuan
	pi	Proportion of primary industry GDP	GDP of primary industry / regional GDP	%
	rpg	Per capita agricultural GDP	Regional agriculture, forestry, animal husbandry and fishery GDP / regional agricultural population	RMB 10000 yuan / person
	ig	Income gap between urban and rural areas	Disposable income of urban residents – disposable income of rural residents	RMB yuan
	lfe	Local financial expenditure on agriculture, forestry and water affairs	Yearbook direct access	RMB million yuan
	cgo	Per capita grain output	Yearbook direct access	kg
	wse	Water saving irrigation efficiency	Water saving irrigation area / grain sowing area	%

### Model Setting

On the basis of the previous research studies, this article refers to the modified C-D production function form of Beck et al. (63) and Zhou et al. (64), and introduces the development level of agricultural insurance, per capita net income of rural residents, the proportion of GDP of primary industry, per capita agricultural GDP, urban–rural income gap, local financial expenditure on agriculture, forestry, and water affairs and per capita grain output into the model as production factor inputs, so the basic model is set as follows:


(7) $$\begin{array}{l} \ln agdi_{it}=\alpha +\beta_{1}\ln ai_{it}+\beta_{2}\ln pi_{it}+\beta_{3}\ln rpg_{it}+\beta_{4}\ln ig_{it}\\ \;+\beta_{5}\ln lfe_{it}+\beta_{6}\ln cgo_{it}\text{+}\beta_{7}\ln{\text{wse}}_{it}\text{+}\lambda_{t}+\mu_{i}+\varepsilon_{it} \end{array}$$

Where the small and medium marks *i* represents different provinces, municipalities, and autonomous regions, *i* = 0, 1, ..., 31; *t* stands for time, *t* = 2008, 2009, …, 2019. lnagdi_it_ is the explained variable, which is the logarithmic form of the agricultural green development index calculated above. lnai_it_ is the variable concerned in this article, which is the logarithmic form of the development level of agricultural insurance. lnri_it_, lnrpg_it_, lnig_it_, lnlfe_it_, lncgo_it_ and lnwse_it_ are control variables, see Table 5 for details. λ_*t*_ and μ_*i*_ are time fixed effect and individual fixed effect, respectively.is the model error term.

## Empirical Results Analysis

### Full Sample Regression Analysis

To analyze the overall impact of agricultural insurance on the level of agricultural green development, according to the model setting, this study used the fixed effect on analysis, and the regression results are shown in Table 6. Column (1) is the regression result of the core explanatory variable, and column (2) is the regression result after adding the control variable. After adding the control variable, the determination coefficient (R-sq) is strengthened, indicating that the addition of the control variable is conducive to the fitting of the model. The empirical results show that the coefficient of the core explanatory variable agricultural insurance development level is negative and significant at the 1% confidence level, which indicates that agricultural insurance development has an inhibitory effect on agricultural green development. This result verifies hypothesis H1B. When the development level of agricultural insurance increases by 1%, the agricultural green development index decreases by 0.0,518%. When the control variable is added, the agricultural green development index decreases by 0.0,378% for every 1% increase in the development level of agricultural insurance. Although the coefficient value is relatively small, it still shows that the current development of agricultural insurance has a crowding-out effect on agricultural green development. The main reason may be that agricultural insurance can stabilize farmers' income expectations, encourage farmers to expand the scale of planting and breeding, and then lead to the transformation of production mode, resulting in the change of chemical application by farmers. In addition, although China's agricultural insurance has developed rapidly, and the premium income of agricultural insurance has increased from RMB 5.33 billion yuan in 2007 to RMB 81.493 billion yuan in 2020 (3), the types of agricultural insurance are single and mostly cost insurance, and the guarantee level of agricultural insurance still needs to be further improved. Taking 2020 as an example, the amount of risk protection provided by agricultural insurance in that year was RMB 4.13 trillion yuan, <30% of the total output value of agriculture, forestry, animal husbandry, and fishery that year (China's total output value of agriculture, forestry, animal husbandry, and fishery in 2020 was RMB 13.778 trillion yuan).

**Table 6:** Full sample regression results.

**Explanatory variable**	**Explained variable lnagdi**	
	**(1)**	**(2)**
ln*ai*	−0.0,518***(0.0,117)	−0.0,378***(0.0,130)
ln*pi*		0.1,515(0.1,358)
ln*rpg*		0.1,001(0.0,953)
ln*ig*		−0.2,884***(0.0,941)
ln*lfe*		0.0,330(0.0,490)
ln*cgo*		−0.1,702**(0.0,807)
ln*wse*		0.0,2749***(0.0,984)
cons	−1.279***(0.0,122)	2.6391**(1.1017)
N	403	403
R–sq	0.2,684	0.3,910

^* *^^*^, ^*^^*^, and ^*^ are significant at the level of 1, 5, and 10%, respectively, and the standard error is expressed in parentheses.

### Regional Regression Results Analysis

The zonal regression in this article is analyzed according to the three production functional areas divided in the outline of the medium and long-term plan for national food security (2008 ~ 2020). The regression results are shown in Table 7. The regression results of each functional area are: (1) the regression results listed as the core explanatory variables, and (2) the regression results listed after adding the control variables. The empirical results show that in the main grain-producing areas and grain production and sales balance areas, the coefficient of the core explanatory variable agricultural insurance development level is negative, and is significant at the confidence level of 1% and 10%. After adding the control variable, the decisive coefficient (R-sq) is strengthened, indicating that the addition of the control variable is conducive to the fitting of the model, and the coefficient symbol of the core explanatory variable has not changed and is still negative. This shows that the development of agricultural insurance has a restraining effect on the green development of agriculture in these two areas. In the main grain sales area, the regression result of the core explanatory variable agricultural insurance development level is not significant, and it is still not significant after adding the control variable. The zonal regression results verify the hypothesis H2A, that is, the impact of agricultural insurance on agricultural green development has regional heterogeneity.

**Table 7:** Partition regression results.

**Explanatory variable**	**Explained variable lnagdi**					
	**Main grain producing areas**		**Main grain sales area**		**Grain production and sales balance area**	
	**(1)**	**(2)**	**(1)**	**(2)**	**(1)**	**(2)**
Lnai	−0.0,8234***	−0.0,352* (0.0,166)	−0.0,407 (0.0,334)	−0.0,960 (0.05,018)	−0.0,342* (0.1,598)	−0.0,318* (0.0,170)
Lnpi		0.1,210 (0.1,193)		0.0,729 (0.0,779)		0.1,354 (0.2,400)
Lnrpg		0.0,751 (0.1,024)		−0.0,563 (0.1,626)		0.2,741* (0.1,420)
Lnig		−0.5,142*** (0.0,896)		0.0,294 (0.1,267)		−0.3,257*** (0.1,074)
Lnlfe		0.1,333** (0.0,460)		0.1,034 (0.0,854)		−0.0,716 (0.0,911)
Lncgo		−0.2,735* (0.1,475)		0.1,105* (0.0,543)		0.0,334 (0.0,795)
Lnwse		0.2,495** (0.1,130)		0.5,226*** (0.0,956)		0.2,150 (0.2,212)
cons	−1.3048***	4.889*** (1.429)	−0.0,990*** (0.03,030)	−1.703783 (0.1,837)	−1.434*** (0.0,232)	2.078 (1.3,559)
N	169	169	91	91	143	143
R–sq	0.4,664	0.6,156	0.1,311	0.4,143	0.2,006	0.3,318

^* *^^*^, ^*^^*^, and ^*^ are significant at the level of 1, 5, and 10%, respectively, and the standard error is expressed in parentheses.

## Conclusions and Suggestions

In this articles, we used the fixed effect model to study the impact of agricultural insurance on agricultural green development, and discussed the regional heterogeneity.

Taking the provincial panel data of China from 2007 to 2019 as the research sample, this article constructs an index system to calculate the agricultural green development index, and then makes an empirical study on the impact of agricultural insurance on agricultural green development. The main conclusions are as follows:

(1) We provide a new idea. Different from previous studies, we studied the overall impact of agricultural insurance on agricultural green development, rather than the impact of agricultural insurance on a chemical input (65), or the increase of input caused by evaluating the income effect of agricultural insurance (30).
(2) From the empirical results of all samples, this article used the fixed effect model to analyze. The empirical results showed that when the development level of agricultural insurance increased by 1%, the agricultural green development index decreased by 0.0,518%. When the control variable was added, the agricultural green development index decreased by 0.0,378% for every 1% increase in the development level of agricultural insurance. Although the coefficient is very small, agricultural insurance has an inhibitory effect on agricultural green development. Our research results are similar to that of Horowitz and other scholars (11, 36–42, 66). In their research, they found that there is a positive correlation between agricultural insurance and chemical input. It is also that agricultural insurance can improve the application intensity of chemicals, and the increase of chemical input is not conducive to the green development of agriculture. However, it is in contrast to the scholars who have a negative correlation between agricultural insurance and chemical investment (17, 33, 67).
(3) This article fully considers the heterogeneity problem. From the regional empirical results, there are regional differences in the impact of agricultural insurance on agricultural green development (26). There are differences in agricultural production environment and breeding habits in different regions, which may lead to such differences. The empirical results show that in the main grain-producing areas and grain-balanced areas, the coefficient of the core explanatory variable agricultural insurance development level is negative, indicating that agricultural insurance has a crowding-out effect on agricultural green development. In the main grain sales areas, this impact is not significant. The estimated results are similar to those of Weber et al. (68). They studied the federal crop insurance in 2000 to 2013. The estimated results show that the expansion of coverage has little impact on farmland harvest share, crop specialization, productivity, or fertilizer and chemical use.

In addition, China's agricultural green development refers to that during 2007–2019, the changing trend of China's three major functional areas' agricultural green development index is similar, and generally presents an approximate “m” -shaped fluctuation change. Although there are differences in the index system, index selection, and research intervals, the evaluation results are similar to the research results of studies in Refs (69, 70). Specifically, the ranking of agricultural green development index: main grain sales areas > main grain production areas > grain balance areas.

The research methods and conclusions of this article are expected to provide some valuable references for the reform and development of agricultural insurance in developing countries. However, as this article uses panel data at the provincial level in China, there may be some inadequacies. In the future, we will most likely collect the panel data of cities and counties in China for analysis, which has supplemented and verified the research conclusions.

Based on the above conclusions, this article puts forward the following suggestions:

(1) Build a low-carbon subsidy mechanism for agricultural insurance. Farmers are guided to make decisions on agricultural production through low-carbon insurance premiums and other measures. Through policy making, farmers will gradually transform the traditional agricultural farming mode with high energy consumption into a low-carbon advanced modern agricultural farming mode with ecological and environmental protection.
(2) Enrich agricultural insurance products and improve the coverage of agricultural insurance. At this stage, China's agricultural insurance is mainly materialized cost insurance, and the guarantee level of agricultural insurance still has scope for improvement (71). Insurance companies can develop some income and index agricultural insurance products to improve the level of agricultural insurance protection. In addition, insurance companies add ecological considerations to the pricing and evaluation of financial insurance (72) to enhance the role of agricultural insurance in the ecosystem. At the same time, with the help of the media, carry out agricultural insurance publicity, improve farmers' insurance awareness, and further expand the coverage of agricultural insurance.
(3) Through policy incentives to build agricultural production mode of combination of planting and breeding and recycling (37).

Encourage straw returning to the field, and some straw can be tried to be converted into feed needed by the breeding industry. Farmers are encouraged to use organic fertilizers and reduce the use of chemicals, and different preferential policies are given according to the proportion of using organic fertilizers. Farmers are encouraged to process livestock manure into organic fertilizer, and to recycle the waste obtained between planting and breeding.

## Data Availability Statement

The original contributions presented in the study are included in the article/supplementary material, further inquiries can be directed to the corresponding author.

## Author Contributions

DH and XW: conceptualization, writing—original draft preparation, and funding acquisition. DH: methodology, software, investigation, and data curation. XW: writing—review and editing. Both authors contributed to the article and approved the submitted version.

## Funding

This research was funded by the Fujian Provincial Social Science Planning Project (Grant No. FJ2021BF038), the Fujian Innovation Strategy Research Project (Grant No. 2021R0075), the National Social Science Foundation of China (Grant No. 20XJY011), the Minnan Normal University President's Fund Project (Grant No. SK18017), the Educational Research Project for Young and Middle-aged Teachers in Fujian Province (Grant No. JAT190735, JAT200311), the Longyan University Startup Foundation for PhD (Grant No. LB2020001), the Fujian Natural Science Foundation (Grant No. 2020J05204), and the Technological Innovation Fund Project of Scientific and Technological Small and Medium-sized Enterprises in Fujian Province (Grant No. 2021C0054).

## Conflict of Interest

The authors declare that the research was conducted in the absence of any commercial or financial relationships that could be construed as a potential conflict of interest.

## Publisher's Note

All claims expressed in this article are solely those of the authors and do not necessarily represent those of their affiliated organizations, or those of the publisher, the editors and the reviewers. Any product that may be evaluated in this article, or claim that may be made by its manufacturer, is not guaranteed or endorsed by the publisher.

## References

[1] Zhang NM Zhang L Zhao H Han YC Duan YH. Construction and application of evaluation index system for agricultural green development. Ecol Econ° (2018) 34:21–4.

[2] Yin CB Li FD Wang S Hao AB. The concept, connotation and principle of China's agricultural green development. J Agric Resour. (2021) 42:1–6.

[3] Zhu JS. Boosting rural revitalization strategy with high-quality development of agricultural insurance - review and prospect of agricultural insurance market in 2020. In: Tuo GZ, editor. Research on Agricultural Insurance in China 2021. Beijing: China Agricultural Press (2021). p. 1–23.

[4] Ma JJ Cui HY Wu BJ. Analysis on the increasing effect and action path of policy agricultural insurance extension on farmers' income– A quasi natural experimental study on the gradual pilot. J Risk Insur. (2020) 2:3–18. 10.13497/j.cnki.is.2020.02.001

[5] Xu TT Sun R. Could policy-oriented agricultural insurance alleviate poverty vulnerability? –*Analysis based on survey data of typical villages Agric Economy*. (2022) 02:126–44. 10.13246/j.cnki.jae.2022.02.005

[6] Agbenyo W Jiang YS Ntim GA. Impact of crop insurance on cocoa farmers' income: an empirical analysis from Ghana. Environ Sci Pollut Res. (2022). 10.1007/s11356-022-20035-1 35397028

[7] Jiang SZ Zhu WC. Does agricultural insurance help to ensure national food security?. Insurance research. (2021) 10:3-17. 10.13497/j.cnki.is.2021.10.001

[8] Feng WL Su XP. On the development of agricultural insurance in the strategy of rural revitalization. Research on Rural Finance. (2019) 4:14–8.

[9] Goodwin BK Vandeveer ML Deal JL. An empirical analysis of acreage effects of participation in the federal crop insurance program. Am J Agric Econ. (2004) 86:1058–77. 10.1111/j.0002-9092.2004.00653.x

[10] Jerry S. The Potential influence of risk management programs on cropping decisions. Presented at ERS Conference. (2000).

[11] Horowitz J K Lichtenberg E. Insurance, moral hazard, and chemical use in agriculture. Am J Agric Econ. (1993) 75:926–35. 10.2307/124398015572077

[12] Smith VH Goodwin BK. Crop Insurance, moral hazard, and agricultural chemical use. Am J Agric Econ. (1996) 78:428–38. 10.2307/124371415572077

[13] Tjalling CK Montias JM. On the description and comparison of economic systems. In: Eckstein A, editor. Comparison of Economic Systems: Theoretical and Methodological Approaches. Oakland, CA: University of California Press (1971). p. 35.

[14] Miranda MJ. Area-yield crop insurance reconsidered. Am J Agric Econ. (1991) 73:233–42. 10.2307/1242708

[15] Akinrinola OO Okunola AM. Evaluation of effects of agricultural insurance scheme on agricultural production in Ondo State. RJOAS. (2014) 28:3–8. 10.18551/rjoas.2014-04.01

[16] Liu F Li HY Gong CG. The effect of agricultural insurance on agricultural output and its heterogeneous factors – An empirical study based on prefecture level cities in Henan Province. J Off Stat. (2020) 36:159–62. 10.13546/j.cnki.tjyjc.2020.21.032

[17] Li QY Chen K Chen LP. Impact of crop insurance participation behavior on rural households' input tendency of chemical elements: a comparative study based on different policy recognition scenarios. J Agric Econ. (2020) 19:280–7. 10.16195/j.cnki.cn36-1328/f.2020.03.30

[18] Dai N Tao JP. An empirical study on the effect of policy-supported agricultural insurance on agricultural production: Based on quantile regression of panel data of 31 provinces in China. J South China Agric Univ. (2017) 22:163–73. 10.11841/j.issn.1007-4333.2017.12.20

[19] Ma SZ Liu MH. Does agricultural insurance promote agricultural productivity? –* Empirical test based on China's inter provincial panel data J Zhejiang Univ Sci B*. (2016) 46:131–44. 10.3785/j.issn.1008-942X.CN33-6000/C.2015.10.193

[20] Yan H Tan D. Analysis on the effect of policy agricultural insurance on agricultural output –a case study of Hubei Province. Rural Econ. (2017) 9:94–100.

[21] Robert GC John Q. Decomposing input adjustments under price and production uncertainty. Am J Agric Econ. (2001) 83:20–34. 10.1111/0002-9092.00134

[22] Porrini D Fusco G Miglietta PP. Post-adversities recovery and profitability: the case of Italian farmers. Int J Environ Res Public Health. (2019) 16:3189. 10.3390/ijerph16173189 31480521 PMC6747205

[23] Ramasubramanian JA. Demand and Impact of Crop Microinsurance In India. Economics PhD Theses. (2015).

[24] Xu B Sun R. Effects of agricultural insurance on farmers' production behavior under the background of grain security: evidence from grain production areas. FinancEcon. (2016) 6:97–111.

[25] Zhang XG Zhao YF. A theoretical and empirical study on the impact of dairy cattle insurance program on the scale of dairy farming. J Risk Insur. (2017) 2:40-49.

[26] Chen JC Wang HM. Agricultural insurance and agricultural non-point source pollution: influencing factors and measurement – scenario simulation based on simultaneous equations model. JSUFE. (2015) 17:34–43. 10.16538/j.cnki.jsufe.2015.05.001

[27] Liang C He J Tao JP. Does agricultural insurance promote land transfer? –Based on the empirical analysis of the three provinces of central China. World Agric. (2022) 1:87–98. 10.13856/j.cn11-1097/s.2022.01.008

[28] Li Y. Influence of policy planting insurance on Farmers' planting behavior. J Inner Mongolia Teach Univ. (2020). 10.27229/d.cnki.gnmnu.2020.000069

[29] Roll KH. Moral hazard: the effect of insurance on risk and efficiency. Agric Econ. (2019) 50:367–75. 10.1111/agec.12490

[30] Hill RV Kumar N Magnan N. Ex ante and ex post effects of hybrid index insurance in Bangladesh. J Dev Econ. (2018) 136:1–17. 10.1016/j.jdeveco.2018.09.003 31007349 PMC6472668

[31] Falco S DF. Adinolfi, Bozzola M, Capitanio F. Crop Insurance as a strategy for adapting to climate change J Agric Econ. (2014) 65:485–504. 10.1111/1477-9552.12053

[32] Wu JJ. Crop insurance, acreage decisions, and nonpoint-source pollution. Am J Agric Econ. (1999) 81:305–20. 10.2307/1244583

[33] Zhang C Lv KY Cheng XY. Dose agricultural insurance affect farmers' application of pesticides? Empirical evidence from grain farmers in 4 provinces J China Agric Univ. (2019) 24:184–94.

[34] Zhang ZX Mu YY Hou LL. Can participating in agricultural insurance optimize the allocation of factors? –*Analysis of endogenous production effect of farmers' insurance behavior China Agric Econ Rev*. (2018) 10:53–70.

[35] Zhong FN Ning MX Xing L Miao Q. Study on the relationship between agricultural insurance and agrochemical application – An empirical analysis of farmers in Manas River Basin, Xinjiang. Economics. (2007) 1:291–308.

[36] Chakir R Hardelin J. Crop insurance and pesticides in French agriculture: an empirical analysis of multiple risks management. Eur Rev Agric Econ. (2010) 27–8.

[37] Luo XM Zhang W Tang Y. Environmental effect of policy agricultural insurance and green subsidy model. Rural Eco. (2016) 11:13–21.

[38] Pan D. The impact of agricultural extension on farmer nutrient management behavior in Chinese rice production: a household-level analysis. Sustainability. (2014) 6:6644–65. 10.3390/su6106644

[39] Niu ZH Feng Y Chen C. Agricultural insurance and agricultural fertilizer non-point source pollution: evidence from China's policy-based agricultural insurance pilot. Sustainability. (2022) 14:1–16. 10.3390/su14052800

[40] Li T Chen LH Li XX Li S Chen HB Ji H. The impact of cost-of-production insurance on input expense of fruit growing in ecologically vulnerable areas: Evidence from Shaanxi province of China. Sustainability. (2021) 13:1–14. 10.3390/su132112083

[41] Capitanio F Adinolfi F Santeramo F. Crop insurance subsidies and environmental externalities: Evidence from Southern Italy. Outlook Agric. (2014) 43:1–100. 10.5367/oa.2014.0183

[42] Wong HL Wei X Kahsay HB Gebreegziabher Z Gardebroek C Osgood DE. Effects of input vouchers and rainfall insurance on agricultural production and household welfare: experimental evidence from northern Ethiopia. World Dev. (2020) 135:105074. 10.1016/j.worlddev.2020.105074

[43] Stein D. Rainfall index insurance in India. J Social, Polit Econ Stud. (2011) 21–5.

[44] De F Vargas NR Robles HM. Interplay Among Credit, Weather Insurance and savings for Farmers in in Developing Countries. Presentation at the American Economic Association Meetings. (2013).

[45] Farrin K Miranda MJ. A heterogeneous agent model of credit-linked index insurance and farm technology adoption. J Dev Econ. (2015) 116:199–211. 10.1016/j.jdeveco.2015.05.001

[46] Brick KI Visser M. Risk preferences, technology adoption and insurance uptake: a framed experiment. J Econ Behav Organ. (2015) 118:383–96. 10.1016/j.jebo.2015.02.010

[47] Miao R. Climate, insurance and innovation: the case of drought and innovations in drought-tolerant traits in US agriculture. Eur Rev Agric Econ. (2020) 5:5. 10.1093/erae/jbaa010

[48] Tan YJ Zhang H. Does contract farming promote the demand of new agricultural operators for agricultural technology? Rural Econ. (2021) 7:129–35.

[49] Ma JJ Cui HY. Carbon emission reduction effect of agricultural insurance development: effect and mechanism. Chin J Popul Resour Environ. (2021) 31:79–89.

[50] Tang YM Yang Y Ge JH. Can “bancassurance interaction” promote farmers' technology adoption? –*Empirical analysis based on field experiment China Rural Econ*. (2019) 1:127–42.

[51] Michael RC Lan C Alexandros S. Where and how index insurance can boost the adoption of improved agricultural technologies. J Dev Econ. (2016) 118:59–71. 10.1016/j.jdeveco.2015.08.008

[52] Liu Y. The impact of agricultural insurance on the adoption of environmentally friendly agricultural technologies. J Agric Sci. (2021).

[53] Schoengold K Ding Y Headlee R. The impact of AD HOC disaster and crop insurance programs on the use of risk-reducing conservation tillage practices. Am J Agric Econ. (2015) 97:897–919. 10.1093/ajae/aau073

[54] Tang YM Yang Y Ge JH Chen J. The impact of weather index insurance on agricultural technology adoption evidence from field economic experiment in China. China Agricultural Economic Review. (2019)11:622-641. 10.1108/CAER-05-2018-0107

[55] Fei YH. The deep root of China's agricultural insurance development dilemma – Based on the analysis of welfare economics. Financial Research. (2005) 3:133–44.

[56] Miglietta PP Porrini D Fusco G Capitanio F. Crowding out agricultural insurance and the subsidy system in Italy: Empirical evidence of the charity hazard phenomenon. Agric Financ Rev. (2020) 81:237–49. 10.1108/AFR-04-2020-0061

[57] Hou DN Wang X. Measurement of agricultural green development level in the three provinces of Northeast China under the background of rural vitalization strategy. Front in Public Health. (2022) 10:824202. 10.3389/fpubh.2022.824202 35359758 PMC8962617

[58] Pourjavad E Shahin A. A hybrid model for analyzing the risks of green supply chain in a fuzzy environment. J Ind Prod Eng. (2020) 37:422–33. 10.1080/21681015.2020.1833995

[59] Tseng ML Tran TPT Ha HM Bui TD. Lim MK. Sustainable industrial and operation engineering trends and challenges toward industry 40: a data driven analysis. J Ind Prod Eng. (2021) 1:581–98. 10.1080/21681015.2021.1950227

[60] Zou XY Kang XY. Research on the poverty reduction effect of agricultural insurance from the perspective of relative poverty – Based on the panel data of 30 provinces (autonomous regions and cities) in China from 2007 to 2019. Contemp Econ Policy. (2022) 5:1–19.

[61] Li QY Chang H Tang HC. The synergy effect of agricultural insurance and agricultural total factor productivity on agricultural output. J. Henan Agric Univ. (2022) 56:143–52. 10.16445/j.cnki.1000-2340.20210412.001

[62] Zhou WH Zhao GL Yin CY. An empirical study on the impact of agricultural insurance on agricultural production —— Based on panel data and dynamic difference GMM model in Hebei Province. J Risk Insur. (2015) 5:60–8. 10.13497/j.cnki.is.2015.05.007

[63] Beck T Asli DK Levine R. Finance, inequality and the poor. J Econ Growth. (2007) 12:27–49. 10.1007/s10887-007-9010-6

[64] Zhou WH Zhao GL Yin CY. Dynamic study on the impact of agricultural insurance development on Farmers' income – An empirical test based on panel system GMM model. J Risk Insur. (2014) 5:21–30. 10.13497/j.cnki.is.2014.05.009

[65] Goodwin BK Smith VH. The effects of crop insurance and disaster relief programs on soil erosion: the case of soybeans and corn. Am J Agric Econ. (1997) 79:1703–4. 10.1007/978-94-017-2915-4_12

[66] Agnieszka KK Agnieszka SR Joanna PT Michał S. Crop Insurance, land productivity and the environment: a way forward to a better understanding. Agriculture. (2021) 11:1108. 10.3390/agriculture11111108

[67] Feng SZ Han YJ Qiu HG. Does crop insurance reduce pesticide usage? Evidence from China China Economic Review. (2021) 69:101679. 10.1016/j.chieco.2021.10167932500500

[68] Weber J Key N O'Donoghue E. Does federal crop insurance make environmental externalities from agriculture worse? J Assoc Environ Resour. (2016) 3:707–42. 10.1086/687549

[69] Guo HP Xu S Pan CL. Measurement of the spatial complexity and its influencing factors of agricultural green development in China. Sustainability. (2020) 12:1–18. 10.3390/su1221925935136666

[70] Zhang XY Chen H. Green agricultural development based on information communication technology and the panel space measurement model. Sustainability. (2021) 13:1–16. 10.3390/su13031147

[71] China Agricultural Insurance Guarantee Level Research Group. Research Report on the Level of Agricultural Insurance in China. Beijing: China Finance Press (2017). p. 25–28.

[72] Valente D Miglietta PP Porrini D Pasimeni MR Zurlini G Petrosillo I. first analysis on the need to integrate ecological aspects into financial insurance. Ecol Modell. (2019) 392:117–27. 10.1016/j.ecolmodel.2018.11.009

